# Community-Level Awareness of Proper Immediate Steps for Ocular Chemical Injury in Saudi Arabia

**DOI:** 10.7759/cureus.45120

**Published:** 2023-09-12

**Authors:** Medhat Taha, Abdulwahab Abdulaziz Alzubaidi, Raneem AlHajri, Renad Albusaad, Rawabi Aljumaiah, Alreem S Aldwsri

**Affiliations:** 1 Department of Anatomy, Umm Al-Qura University, Makkah, SAU; 2 Department of Medicine, Umm Al-Qura University, Makkah, SAU; 3 Department of Medicine, Arabian Gulf University, Manama, BHR; 4 Department of Neurology, King Faisal University, Al Hofuf, SAU; 5 Department of Medicine, King Faisal University, Al Hofuf, SAU

**Keywords:** community, complications, saudi arabia, awareness, ocular chemical injury

## Abstract

Background: The human eyes are the most vital sensory organ. Eye injury is the main factor leading to monocular blindness. There is a lack of understanding in the Saudi community regarding the appropriate response to a chemical injury to the eyes. To provide accurate and clear medical information about ocular chemical damage, it is crucial to identify areas where healthcare professionals fall short. To address the gaps in critical knowledge, this study aimed to focus on the Saudi Arabian community to determine if healthcare professionals have fulfilled their responsibility as health promoters in providing education on eye emergencies. We examined the immediate reaction of the study participants to evaluate the community's awareness of chemical eye injuries and immediate management strategies.

Objectives: This study aimed to assess and evaluate the general population's knowledge and awareness of immediate management steps following an ocular chemical injury in Saudi Arabia.

Methodology: A cross-sectional design was employed. A random sampling method was used to select 2,295 individuals from the Saudi community. Participants completed an electronic closed-ended, pre-validated, anonymous, and self-administered questionnaire.

Results: The majority of the respondents were females (71.6%) and Saudis (94.5%) aged 18 to 30 years (46.6%). The majority of the respondents correctly stated that eye injuries could cause complications (89.6%) and that common substances that could result in eye injuries are chloride and detergents (93.3%) and battery materials (73.1%). Furthermore, complications after eye injuries could include blindness (80.6%), perforation (44.5%), and scar formation (38.1%). A substantial proportion of the respondents indicated that water should be used to treat chemical injuries due to acidic and alkaline substances (68.1%). However, only a small proportion of the respondents correctly answered that the eyes should be washed for 30 minutes or more after an ocular injury (7.9%).

Conclusions: The overall knowledge levels on injury mechanisms and complications were generally high with a lack of knowledge regarding immediate management for injuries. Therefore, there is a need for targeted educational interventions and training programs to enhance the general public’s understanding of immediate management strategies for chemical eye injuries.

## Introduction

The human eyes are the most vital sensory organ. In the United States, eye injury is the main causative factor leading to monocular blindness, ranking second only to cataracts as the most common cause of vision impairment [1]. Eye injury is the most common reason for people to visit emergency departments for eye-related issues [2]. A chemical eye injury is a critical ophthalmic emergency that requires urgent clinical evaluation and treatment [3]. One or both eyes may lose vision as a result of chemical eye injuries such as the cornea, ocular surface epithelium, limbal stem cells, and anterior segment could be severely damaged [4]. In cases of a chemical burn, a liquid or powder chemical comes into contact with the eyes. The injury typically occurs when a chemical splashes on the face as well as when wiping the eyes after using chemicals. Alkaline and acidic injuries are both types of chemical injuries. Due to the extensive use of alkaline chemicals in household and industrial cleansers, alkaline burns are more common and cause severe injuries [5]. A prior study in Saudi Arabia examined chemical injury data from two important state-run facilities. Alkaline drain cleaners accounted for 75% of all cases of chemical burns among 59 patients (male-to-female ratio, 3:1; mean age, 25 years). In the remaining cases, causes of eye injuries included topical use of herbs, concentrated sulfuric acid, and automotive battery acid [6]. It has been reported that 30%, 60%, and 10% of chemical injuries occur at home, at work, and following an accident, respectively [7]. Up to 20% of chemical injuries cause severe visual impairment and facial deformity; notably, less than 15% of those with advanced chemical eye injuries experience visual recovery. Chemical injuries are three times more common among men than among women, and they most commonly affect people between the ages of 16 and 45.7 years. In Saudi Arabia, a study found that people need to be more aware of the importance of timely corrective steps in cases of ocular chemical damage [8]. There is a lack of understanding in the Saudi community regarding the appropriate response to a chemical injury to the eyes. To provide accurate and clear medical information on ocular chemical damage, it is crucial to identify areas where healthcare professionals fall short. To address the gaps in critical knowledge, this study aimed to focus on the Saudi Arabian community to determine if healthcare professionals have fulfilled their responsibility as health promoters in providing education on eye emergencies. We examined the immediate reaction of the study participants to evaluate the community's awareness of chemical eye injuries and immediate management strategies.

## Materials and methods

A cross-sectional study involving Saudi Arabian citizens and residents was conducted in March 2023. A random sampling method was utilized to select 2,295 participants. Those who participated completed a pre-validated questionnaire used in a previous study [9]. Permission was obtained from the corresponding author in the English language. The electronic questionnaire contained closed-ended, anonymous, and self-administered questions covering demographic data, history of ophthalmic injuries, and employment details (medical-related or other), as well as awareness-related questions. It was distributed through several social media platforms with the help of data collectors from different regions in Saudi Arabia to ensure data accuracy. The study included both males and females ≥18 years of age. Ethical approval was obtained from the Biomedical Research Ethics Committee of Umm Al-Qura University (Al-Qunfudhah, Saudi Arabia, approval number HAPO-02-K-012-2023-04-1590). The sample size was determined using a sample size calculator (http://www.raosoft.com/ samplesize.html); the minimum recommended sample size was 380, and we included 2,295 respondents. Permission was obtained from the participants before administering the questionnaire, and they were informed about the estimated duration for completing the questionnaire. Furthermore, the purpose of the study was explained and presented with the link for the online questionnaire.

Data analysis and scoring

Datasets were extracted, revised, coded, and analyzed using IBM SPSS® Statistics version 22 (IBM Corp. Released 2013. IBM SPSS Statistics for Windows, Version 22.0. Armonk, NY: IBM Corp.). An overall knowledge score was calculated based on a total of 17 questions with a correct response. Each correct response was assigned a score of 1, whereas other responses were assigned a score of 0. The knowledge score was computed by summing up the relevant correct answers. Therefore, the scores ranged from 0 and 17. Normality testing was applied to the knowledge score, and the results indicated a non-normally distributed variable (Shapiro-Wilk test, p<0.0001). Therefore, numerical data are presented as the median and IQR. Statistical differences in knowledge scores were assessed by the Wilcoxon rank-sum test for variables with two categories and the Kruskal-Wallis rank-sum test for variables with three or more categories. Independent predictors of high knowledge scores were assessed by a general linear model using significant variables from bivariate analysis, and the knowledge score was incorporated as a dependent variable. Statistical significance was set at p<0.05.

## Results

Data from a total of 2,295 participants were received on the online platform. However, we excluded 30 records from respondents who did not agree to participate. Therefore, 2,265 records were analyzed in the current study. The majority of the respondents were females (71.6%) and Saudis (94.5%) and had obtained a bachelor’s degree (65.5%). Almost half of the respondents were married (51.7%) and aged 18 to 30 years (46.6%). Employees and students comprised 42.7% and 34.4% of the sample, respectively. A total of 412 respondents had a family history of a chemical eye injury (18.2%). Detailed information on sociodemographic characteristics is presented in Table 1.

**Table 1:** Demographic data

Parameter	Category	N (%)
Age	18-30	1,055 (46.6%)
	31-40	417 (18.4%)
	>40	793 (35.0%)
Gender	Male	643 (28.4%)
	Female	1,622 (71.6%)
Nationality	Non-Saudi	125 (5.5%)
	Saudi	2,140 (94.5%)
Region	Northern region	265 (11.7%)
	Southern region	345 (15.2%)
	Eastern region	476 (21.0%)
	Western region	541 (23.9%)
	Middle region	638 (28.2%)
Educational level	Elementary school	21 (0.9%)
	Middle school	45 (2.0%)
	High school	553 (24.4%)
	Bachelor’s degree	1,484 (65.5%)
	Master’s degree	121 (5.3%)
	PhD degree	41 (1.8%)
Occupation	Student	779 (34.4%)
	Employee	968 (42.7%)
	Retired	171 (7.5%)
	Unemployed	347 (15.3%)
Marital status	Single	1,004 (44.3%)
	Married	1,171 (51.7%)
	Divorced	68 (3.0%)
	Widowed	22 (1.0%)
Have you or any of your relatives had a history of a chemical eye injury?	Yes	412 (18.2%)

The majority of the respondents correctly stated that eye injuries could cause complications (89.6%) and that common substances that could result in eye injuries are chloride and detergents (93.3%) and battery materials (73.1%). Furthermore, problems after eye injuries could include blindness (80.6%), perforation (44.5%), and scar formation (38.1%). Considerable proportions of the respondents indicated that water should be used to treat chemical injuries due to acidic and alkaline substances (68.1%), rubbing the eyes after exposure to chemicals makes the damage to the eyes worse (85.0%), and contact lenses should be removed in cases of ocular injuries (62.8%). In contrast, a small proportion of the respondents correctly answered that the eyes should be washed for 30 minutes or more after an ocular injury (7.9%), treatment options do not depend on the type of ocular injury (5.3%), and the intensity of the pain does not indicate a serious chemical injury to the eyes (11.9%). Detailed information on the participants’ responses is shown in Table 2.

**Table 2:** Participants’ responses to questions for knowledge assessment An asterisk (*) indicates a correct answer.

Parameter	Category	N (%)
Can an eye injury from a chemical cause complications?	I do not know	201 (8.9%)
	Yes*	2,029 (89.6%)
	No	35 (1.5%)
What problems can occur as a result of an eye injury with chemicals?	Blindness*	1,825 (80.6%)
	Cataract	1,416 (62.5%)
	Cancer	442 (19.5%)
	Keratoconus	676 (29.8%)
	Perforation*	1,007 (44.5%)
	Scar*	863 (38.1%)
What substances commonly result in an ocular injury?	Chloride and detergents*	2,114 (93.3%)
	Battery materials*	1,655 (73.1%)
	Water	65 (2.9%)
	Vinegar	956 (42.2%)
What would you do immediately after an ocular chemical injury?	Irrigation of the eyes with a large amount of water*	1,523 (67.2%)
	Go to the emergency department	594 (26.2%)
	Irrigation of the eyes with a small amount of water	76 (3.4%)
	Use eye drops from the pharmacy	34 (1.5%)
	Cover the eyes	38 (1.7%)
What method will you use to treat a chemical eye injury?	An acidic substance if the injury substance is alkaline (e.g., vinegar)	54 (2.4%)
	An alkaline substance if the injury substance is acidic (e.g., soap)	54 (2.4%)
	Water in both cases*	1,542 (68.1%)
	I do not know	615 (27.2%)
Treatment options depend on the type of ocular injury.	I do not know	562 (24.8%)
	Yes	1,582 (69.8%)
	No*	121 (5.3%)
How long should you wash your eyes after an ocular chemical injury?	I do not know	569 (25.1%)
	<5 min	570 (25.2%)
	5-15 min	948 (41.9%)
	30 min or more*	178 (7.9%)
Does the intensity of the pain indicate a serious chemical injury to the eyes?	I do not know	506 (22.3%)
	Yes	1,490 (65.8%)
	No*	269 (11.9%)
Does rubbing your eyes after exposure to chemicals make the damage to your eyes worse?	I do not know	283 (12.5%)
	Yes*	1,925 (85.0%)
	No	57 (2.5%)
What is the most serious sign of an ocular injury?	Eye redness	396 (17.5%)
	Eye white discoloration*	486 (21.5%)
	Eyelid sticking	607 (26.8%)
	Severe pain	776 (34.3%)
Do you think wearing contact lenses can prevent ocular chemical damage?	I do not know	445 (19.6%)
	Yes	206 (9.1%)
	No*	1,614 (71.3%)
If you experience an ocular chemical injury, you should remove contact lenses.	I do not know	635 (28.0%)
	Yes*	1,422 (62.8%)
	No	208 (9.2%)
Do you think that wearing goggles reduces the risk of an ocular injury?	I do not know	208 (9.2%)
	Yes*	1,974 (87.2%)
	No	83 (3.7%)
You should wash your hands after handling chemicals before touching your eyes.	I do not know	100 (4.4%)
	Yes*	2,139 (94.4%)
	No	26 (1.1%)

**Figure 1:**
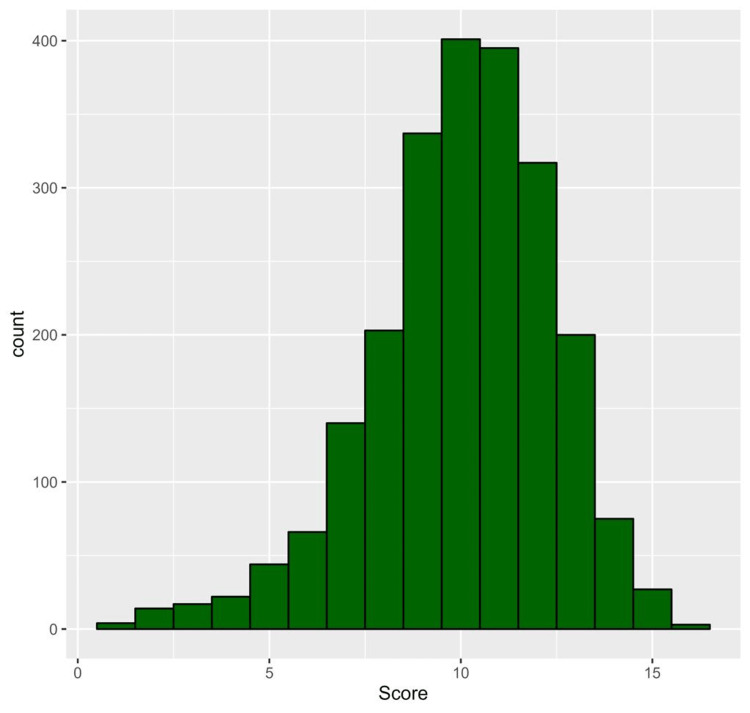
Histogram of the frequency distribution of the knowledge scores of participants

The median (IQR) knowledge score was 10.0 (9.0 to 12.0) with a minimum score of 1 and a maximum score of 16. The distribution of the knowledge score is shown in Figure 1.

Knowledge scores differed significantly according to the participant's age (p<0.001), region of residence (p<0.001), educational level (p<0.001), occupation (p<0.001), and marital status (p< 0.001) (Table 3).

**Table 3:** Differences in knowledge scores according to the demographic characteristics of participants

Parameter	Category	Median (IQR)	p-value
Age	18-30	10.0 (9.0, 12.0)	<0.001
	31-40	10.0 (8.0, 11.0)	
	>40	10.0 (9.0, 11.0)	
Gender	Male	10.0 (9.0, 12.0)	0.105
	Female	10.0 (9.0, 12.0)	
Nationality	Non-Saudi	10.0 (8.0, 12.0)	0.575
	Saudi	10.0 (9.0, 12.0)	
Region	Northern region	10.0 (9.0, 12.0)	<0.001
	Southern region	10.0 (8.0, 11.0)	
	Eastern region	10.0 (9.0, 12.0)	
	Western region	11.0 (9.0, 12.0)	
	Middle region	10.0 (9.0, 11.0)	
Educational level	Elementary school	8.0 (8.0, 12.0)	<0.001
	Middle school	9.0 (7.0, 10.0)	
	High school	10.0 (8.0, 11.0)	
	Bachelor’s degree	10.0 (9.0, 12.0)	
	Master’s degree	10.0 (8.0, 12.0)	
	PhD degree	10.0 (9.0, 12.0)	
Occupation	Student	11.0 (9.0, 12.0)	<0.001
	Employee	10.0 (9.0, 11.0)	
	Retired	10.0 (9.0, 11.0)	
	Unemployed	10.0 (9.0, 11.0)	
Marital status	Single	10.0 (9.0, 12.0)	<0.001
	Married	10.0 (9.0, 11.0)	
	Divorced	10.0 (8.0, 11.0)	
	Widowed	9.0 (8.0, 10.8)	
Have you or any of your relatives had a history of a chemical eye injury?	No	10.0 (9.0, 12.0)	0.156
	Yes	10.0 (9.0, 12.0)	

In regression analysis, residents in the southern region had lower knowledge scores (beta = -0.48, 95% CI: -0.86 to -0.11, p = 0.012), whereas residents in the western region had higher knowledge scores (beta = 0.37, 95% CI: 0.03 to 0.72, p = 0.034). Taking participants whose highest educational level was elementary school as the reference group, the following groups were independently associated with higher knowledge scores: high school (beta = 1.16, 95% CI: 0.12 to 2.20, p = 0.029), bachelor’s degree (beta = 1.71, 95% CI: 0.68 to 2.75, p = 0.001), master’s degree (beta = 1.51, 95% CI: 0.39 to 2.63, p = 0.008), and PhD degree (beta = 1.42, 95% CI: 1.16 to 2.67, p = 0.027). On the other hand, employed participants and retired participants had lower knowledge scores compared with the scores of students (beta = -0.52, 95% CI: -0.86 to -0.17, p = 0.003; beta = -0.54, 95% CI: -1.06 to -0.03, p = 0.038; and beta = -0.49, 95% CI: -0.87 to -0.11, p = 0.012, respectively) (Table 4).

**Table 4:** Predictors of the high knowledge scores of participants

Parameter	Category	Beta	95% CI	p-value
Age	18-30	Ref	Ref	
	31-40	-0.11	-0.49, 0.26	0.560
	>40	0.25	-0.13, 0.63	0.205
Region	Northern region	Ref	Ref	
	Southern region	-0.48	-0.86, -0.11	0.012
	Eastern region	0.20	-0.16, 0.55	0.277
	Western region	0.37	0.03, 0.72	0.034
	Middle region	-0.04	-0.38, 0.29	0.796
Educational level	Elementary school	Ref	Ref	
	Middle school	0.04	-1.18, 1.26	0.952
	High school	1.16	0.12, 2.20	0.029
	Bachelor’s degree	1.71	0.68, 2.75	0.001
	Master’s degree	1.51	0.39, 2.63	0.008
	PhD degree	1.42	0.16, 2.67	0.027
Occupation	Student	Ref	Ref	
	Employee	-0.52	-0.86, -0.17	0.003
	Retired	-0.54	-1.06, -0.03	0.038
	Unemployed	-0.49	-0.87, -0.11	0.012
Marital status	Single	Ref	Ref	
	Married	-0.17	-0.51, 0.17	0.317
	Divorced	-0.44	-1.07, 0.18	0.166
	Widowed	-0.46	-1.53, 0.60	0.397

## Discussion

In the current study, data from a total of 2,295 participants were collected through an online platform. However, 30 records were excluded from the analysis as the respondents withdrew their participation, resulting in a final sample size of 2,265 records for the current study. The sociodemographic characteristics of the participants were examined to gain a comprehensive understanding of the sample. The majority of the respondents in this study were females, comprising 71.6% of the total participants. This distribution suggests a potential gender bias in the sample, with female participants being overrepresented. It is important to acknowledge this limitation as it may affect the generalizability of the study’s findings to the broader population. The study predominantly included participants from Saudi Arabia, accounting for 94.5% of the respondents. This high proportion of Saudi participants may be attributed to the recruitment strategy employed, which may have targeted specific populations or regions within the country. Consequently, the generalizability of the findings to other populations or countries may be limited. In terms of educational background, a significant proportion of the participants had a bachelor’s degree, accounting for 65.5% of the sample. This finding suggests that the study may have attracted a population that was fairly educated, which could influence their knowledge and attitudes toward the topic under investigation. Marital status data revealed that approximately half of the respondents were married (51.7%), and the remaining participants were likely single, divorced, or widowed. The marital status distribution indicates that the sample comprised individuals with different relationship statuses, which might influence their perceptions or experiences related to the study’s subject matter. The age distribution of the participants showed that most of the respondents were 18 to 30 years of age, accounting for 46.6% of the sample. Therefore, the study may have attracted a relatively younger population. Occupationally, the sample comprised a diverse range of individuals, with employees representing the largest group at 42.7% and students accounting for 34.4% of the sample. These findings indicate that the study managed to recruit participants from various professional backgrounds and stages of education. Moreover, a noteworthy finding of this study was that 18.2% of the respondents reported having a family history of a chemical eye injury. Therefore, a considerable proportion of the sample had prior experience or showed familial susceptibility to chemical eye injuries. This finding could have implications for understanding the participants’ perceptions, attitudes, and behaviors toward eye safety practices or related interventions.

Our study assessed the participants’ knowledge regarding eye injuries and their associated complications. The results revealed both areas of strong understanding and areas that require improvement. The majority of the respondents correctly answered that eye injuries can lead to complications, indicating a good overall awareness of this issue (89.6%). Similarly, a high proportion of the participants correctly identified common substances that can cause eye injuries, such as chloride and detergents (93.3%) and battery materials (73.1%). These findings are in agreement with those of previous studies that have highlighted the importance of recognizing potential sources of eye injuries [10,11]. The respondents demonstrated a reasonable level of understanding of potential problems associated with eye injuries. A significant number of participants correctly answered that eye injuries can result in blindness (80.6%), perforation (44.5%), and scar formation (38.1%). This awareness is crucial as it could encourage individuals to avoid the potential severity and long-term consequences of eye injuries. Previous studies have also highlighted these conditions as common complications of eye injuries [12,13]. A considerable proportion of participants believed that water should be used to treat chemical injuries caused by acidic and alkaline substances (67.2%). Proper irrigation water, saline, or specialized eyewash solutions are essential in such cases [14]. Similarly, a high percentage of respondents (85.0%) believed that rubbing the eyes after exposure to chemicals would worsen the damage. Another study highlighted the guidelines on appropriate actions following chemical exposure [4]. Another important finding was that a significant proportion of the participants (62.8%) recognized the need to remove contact lenses in the event of ocular injuries. This is consistent with current guidelines as contact lenses can trap chemicals or foreign bodies and cause further damage to the eyes [15]. However, a low percentage of the participants knew that the eyes should be washed for 30 minutes or more after an ocular injury (7.9%). A previous study has demonstrated that techniques such as extended water irrigation of the eyes post-injury markedly led to reduced hospitalization [16].

Analysis of the factors associated with the participants’ knowledge scores provided valuable insights. The results indicated that age, region of residence, educational level, occupation, and marital status were significantly associated with knowledge scores. Specifically, residents had lower knowledge scores in the southern region compared with other regions, whereas residents had higher scores in the western region. This regional variation suggests disparities in access to eye health education and resources, which should be addressed through targeted interventions and awareness campaigns. Higher educational attainment was associated with higher knowledge scores, with individuals holding a high school degree or higher demonstrating a better understanding. This finding is consistent with that of previous studies that have shown a positive correlation between education level and health knowledge [17]. Efforts to improve eye injury knowledge should focus on individuals with lower educational attainment to bridge this knowledge gap. The occupation of the participants also affected knowledge scores, with employed and retired participants having lower scores compared with the scores of students. This observation suggests that individuals actively engaged in the workforce may have less time or opportunity to stay updated on eye injury prevention and management. Tailored educational programs targeting working populations could help enhance their knowledge and raise awareness. Several previous studies have examined knowledge and awareness related to eye injuries and their management. For example, Dua et al. conducted a similar survey-based study and obtained comparable results regarding common substances causing eye injuries [18].

The consistency of the findings across studies suggests that these findings are robust and representative of the broader population. However, other studies have reported variations in knowledge levels. Alhothali et al. conducted a study and reported a higher overall knowledge score compared to the present study [19]. These differences could be attributed to variations in the study populations, geographical regions, or educational programs implemented to enhance eye injury awareness in specific contexts. Nevertheless, it is important to note that knowledge alone does not necessarily translate into behavior changes or better outcomes. Even individuals with high knowledge scores may not always adhere to recommended practices. Therefore, future research should examine the factors influencing the translation of knowledge into action to improve eye injury prevention and management.

The study has several limitations that should be taken into consideration when interpreting the findings. First, the reliance on self-reported data collected through an online platform raises concerns about response bias and the accuracy of the information provided. Moreover, the sample composition may be a limitation in terms of generalizability. The majority of the respondents were female and Saudi and had obtained a bachelor’s degree, which may not be representative of the overall population. Lastly, the cross-sectional design of the study presents limitations in terms of establishing causal relationships and temporal changes.

## Conclusions

This study highlighted the importance of assessing the awareness and knowledge of the public regarding chemical eye injuries. While overall knowledge levels were generally high, some misconceptions and knowledge gaps were observed among the participants, particularly regarding the duration of eye washing after an injury. Therefore, there is a need for targeted educational interventions and training programs to enhance the general public’s understanding of immediate management strategies for chemical eye injuries. Addressing these knowledge gaps can contribute to improved patient outcomes and reduced vision impairment resulting from such injuries.
